# Activation of Protein Tyrosine Phosphatase Non-Receptor Type 2 by Spermidine Exerts Anti-Inflammatory Effects in Human THP-1 Monocytes and in a Mouse Model of Acute Colitis

**DOI:** 10.1371/journal.pone.0073703

**Published:** 2013-09-09

**Authors:** Belén Morón, Marianne Spalinger, Stephanie Kasper, Kirstin Atrott, Isabelle Frey-Wagner, Michael Fried, Declan F. McCole, Gerhard Rogler, Michael Scharl

**Affiliations:** 1 Division of Gastroenterology and Hepatology, University Hospital Zurich, Zurich, Switzerland; 2 Division of Biomedical Sciences, University of California Riverside, Riverside, California, United States of America; 3 Zurich Center for Integrative Human Physiology, University of Zurich, Zurich, Switzerland; McGill University, Canada

## Abstract

**Background:**

Spermidine is a dietary polyamine that is able to activate protein tyrosine phosphatase non-receptor type 2 (PTPN2). As PTPN2 is known to be a negative regulator of interferon-gamma (IFN-γ)-induced responses, and IFN-γ stimulation of immune cells is a critical process in the immunopathology of inflammatory bowel disease (IBD), we wished to explore the potential of spermidine for reducing pro-inflammatory effects *in vitro* and *in vivo*.

**Methods:**

Human THP-1 monocytes were treated with IFN-γ and/or spermidine. Protein expression and phosphorylation were analyzed by Western blot, cytokine expression by quantitative-PCR, and cytokine secretion by ELISA. Colitis was induced in mice by dextran sodium sulfate (DSS) administration. Disease severity was assessed by recording body weight, colonoscopy and histology.

**Results:**

Spermidine increased expression and activity of PTPN2 in THP-1 monocytes and reduced IFN-γ-induced phosphorylation of signal transducer and activator of transcription (STAT) 1 and 3, as well as p38 mitogen-activated protein kinase (MAPK) in a PTPN2 dependent manner. Subsequently, IFN-γ-induced expression/secretion of intracellular cell adhesion molecule (ICAM)-1 mRNA, monocyte chemoattractant protein (MCP)-1, and interleukin (IL)-6 was reduced in spermidine-treated cells. The latter effects were absent in PTPN2-knockdown cells. In mice with DSS-induced colitis, spermidine treatment resulted in ameliorated weight loss and decreased mucosal damage indicating reduced disease severity.

**Conclusions:**

Activation of PTPN2 by spermidine ameliorates IFN-γ-induced inflammatory responses in THP-1 cells. Furthermore, spermidine treatment significantly reduces disease severity in mice with DSS-induced colitis; hence, spermidine supplementation and subsequent PTPN2 activation may be helpful in the treatment of chronic intestinal inflammation such as IBD.

## Introduction

Inflammatory Bowel Diseases (IBD) is a group of complex, chronic intestinal inflammatory disorders with unknown etiology, including Crohn’s Disease (CD) and ulcerative colitis (UC). IBD patients suffer from recurrent or chronic gastrointestinal symptoms, including diarrhea, abdominal pain, bleeding, anemia and weight loss. Additionally, a spectrum of extraintestinal manifestations, such as joint inflammation, skin and eye manifestations or primary sclerosing cholangitis, may be associated with these diseases. Conventional therapies (*e.g.* aminosalicylates, antibiotics, corticosteroids and immunosuppressants) as well as biologicals (*e.g.* anti-tumour necrosis factor (TNF) and anti-integrin antibodies) are available to treat CD and UC. However, side effects of these treatments are often severe and loss of response over time is common [1], [2], indicating an urgent need for developing novel strategies to treat IBD.

Although the pathogenesis of IBD is not completely understood, a well-accepted hypothesis is that dysregulation of the mucosal immune response against normal intestinal microbiota in genetically susceptible individuals contributes to the development of the disease [3], [4]. Data from genome-wide association studies have identified more than 140 genetic loci that confer susceptibility for IBD [5]. Among them is the gene locus encoding protein tyrosine phosphatase non-receptor type 2 (*ptpn2*) [6], [7], [8]. PTPN2, also known as “T-cell protein tyrosine phosphatase”, is an intra-cellular tyrosine-specific phosphatase, which is expressed in epithelial cells, fibroblasts or endothelial cells featuring particularly high expression in hematopoietic tissues [9]. Two splice variants of PTPN2 are present in human cells: a 48 kDa form which is located in the endoplasmic reticulum, and a 45 kDa variant which is predominantly found in the nucleus. The nuclear variant translocates to the cytoplasm in response to pro-inflammatory stimuli such as interferon-gamma (IFN-γ), epidermal growth factor (EGF), hyperosmotic stress or starvation [10]. Several phosphorylated proteins are well known targets of dephosphorylation by PTPN2, including the epidermal growth factor receptor [11], vascular endothelial growth factor receptor-2 [12], the insulin receptor [13], signal transducers and activators of transcription 1 and 3 (STAT1 and STAT3) [14], [15], and mitogen-activated protein kinase (MAPK)-isoforms, such as p38 [16]. Inactivation of those substrates by dephosphorylation results in the negative regulation of signaling pathways involved in inflammatory responses induced by the pro-inflammatory cytokines IFN-γ [17], [18], TNF [19] and interleukin (IL)-6 [14].

Spermidine is a positively charged polyamine, present in high quantities in foods including mushrooms, peas, cashews, and some types of meat and cereals [20]. Because of its role in cell growth, survival and proliferation [21], [22], spermidine supplementation is being tested to treat conditions in which regeneration and healing are needed, such as impaired liver function, liver resection or burn injury [21], [23]. A role of polyamines in inflammatory responses has also been suggested. Specifically, it has been shown that spermidine is able to increase the phosphatase activity of PTPN2 *in vitro* in multiple cell lines including HeLa cells and human umbilical vein endothelial cells (HUVEC) [24]. Given the reported function of PTPN2 as a negative regulator of pro-inflammatory cytokine signaling [17], [19], [25], we hypothesized that pharmacological activation of PTPN2 by spermidine could potentially ameliorate pro-inflammatory responses induced by cytokines. To test our hypothesis, we first analyzed the effects of spermidine on IFN-γ-induced pro-inflammatory signaling and cytokine production in human THP-1 monocytes. Monocytes/macrophages, as part of the innate immune system of the gut mucosa, play an important role in the pathogenesis of IBD [26]. We found that the activation of PTPN2 by spermidine negatively regulated IFN-γ-induced signaling and cytokine secretion in THP-1 cells, conferring protection against the inflammatory responses induced by this cytokine. Subsequently, the therapeutic effect of spermidine was investigated *in vivo* using an experimental model of colitis. In mice with dextran sodium sulfate (DSS)-induced colitis, disease activity was reduced upon treatment with spermidine, supporting its anti-inflammatory potential as a therapy to treat IBD.

## Methods

### Induction of Colitis and Spermidine Treatment in Mice

Animal experiments were carried out according to Swiss animal welfare laws and were approved by the veterinary authorities of Zurich, Switzerland (Kanton Zürich Gesundheitsdirektion Veterinäramt, approval no. 54/2011). Due to the approval of the veterinary authorities of Zürich, no further approval by an Institutional Animal Care and Use Committee (IACUC) or ethics committee was necessary. Seven- to eight-week-old female C57BL/6J-Crl mice were used for the experiments and housed in a specified pathogen-free facility in individually ventilated cages. Acute colitis was induced with 2.5% DSS (MP Biomedicals, Illkirch, France) in drinking water during 8 days [27]. The animals were randomly divided into two DSS groups and two water control groups with six individuals each. For treatment, spermidine was dissolved in water at 0.1 M and 150 µl administered by oral gavage. The non-treated control groups received 150 µl of water by oral gavage. Food and water were available *ad libitum*.

### Assessment of Colonoscopy and Histological Score in Mice

Animals were anesthetized intraperitoneally with 90–120 mg/kg body weight ketamine (Vétoquinol, Bern, Switzerland) and 8 mg/kg body weight xylazine (Bayer, Lyssach, Switzerland). Animals were examined as described previously [28]. Briefly, the solid endoscope was introduced per anus with a lubricant (2% lidocaine) in the sedated mouse. The colon was gently inflated with air. Recording was performed with the Karl Storz Tele Pack Pal 20043020 (Karl Storz Endoskope, Tuttlingen, Germany). Colonoscopy was scored using the murine endoscopic index of colitis severity (MEICS) scoring system as described previously [28]. In detail, the MEICS score was assessed as follows: The MEICS consisted of five parameters, as indicated: (1) Thickening of the colon (transparent, moderate, marked, non-transparent 0–3 points), (2) changes of the vascular pattern (normal, moderate, marked, bleeding, 0–3 points), (3) fibrin visible (none, little, marked, extreme 0–3 points), (4) granularity of the mucosal surface (none, moderate, marked, extreme 0–3 points) and (5) stool consistency (normal+solid, still shaped, unshaped, spread 0–3 points). The overall score range is then between 0–15. After colonoscopy, all animals were sacrificed by cervical dislocation. Histological scoring for inflammatory infiltration and epithelial cell damage was performed on H&E stained section of the most distal 1 cm of the mouse colon as described previously [27], [28].

### Myeloperoxidase (MPO) Activity Assay

Colon specimens were rinsed with PBS and homogenized mechanically in 50 mM phosphate buffer (pH 6.0) and 0.5% hexadecyltrimethylammonium bromide (Sigma Aldrich) with a tissuelyzer (Qiagen). After three freeze and thaw cycles, homogenates were centrifuged for 2 min at 17,000 g. 20 µl of the supernatant were transferred to a 96-well plate in duplicate and mixed with 280 µl of 0.02% dianisidine (in 50 mM phosphate buffer, pH 6.0, and 0.0005% H_2_O_2_; Sigma Aldrich). After 20 min, absorbance was measured at 460 nm. Protein concentration of the supernatant was determined by bicinchoninic acid protein assay. Myeloperoxidase activity, expressed as arbitrary units, was calculated as mean absorbance (460 nm) per incubation time (in min) per protein concentration (in g).

### Antibodies

The monoclonal mouse anti-PTPN2 antibody CF-4 that detects the 45 kDa and the 48 kDa isoforms and the monoclonal mouse anti-protein tyrosine phosphatase 1B (PTP1B) AE4 antibody were obtained from Calbiochem (San Diego, CA). Mouse anti-β-actin antibody was purchased from EMD Millipore (Billerica, MA). Rabbit anti-phospho-STAT1 (Tyr701), rabbit anti-STAT1, rabbit anti-phospho-STAT3 (Tyr705), rabbit anti-STAT3, mouse anti-phospho-p38 (Thr180/Tyr182) and rabbit anti-p38 antibodies were obtained from Cell Signaling Technologies (Danvers, MA).

### Cell Culture

Human monocytic THP-1 cells were cultured in a humidified atmosphere with 5% CO_2_ in RPMI 1640 medium (Life Technologies Ltd, Paisley, UK) containing 10% fetal calf serum (VWR International, Radnor, PA). Before treatment with recombinant human IFN-γ (1000 U/ml; Roche, Basel, Switzerland) and/or spermidine (100 µM; Merck, Darmstadt, Germany), 5×10^5^ cells were seeded for 2 days in 24-well plates (Techno Plastic Products AG, Trasadingen, Switzerland).

### Preparation of Whole Cell Lysates

After treatment, THP-1 cells were kept on ice. Cells were washed twice with phosphate buffered saline (PBS) and lysed in M-Per Mammalian protein extraction reagent® (Pierce Biotechnology, Rockford, IL) supplemented with the protease inhibitor cocktail tablets “Complete mini®” (Roche) for 30–45 min. Cells were then centrifuged for 10 min at 13,000 g, and supernatants were collected. Protein concentration was quantified by UV_280 nm_ using a NanoDrop ND1000 (Thermo Scientific, Waltham, MA).

### RNA Isolation and Real-time Polymerase Chain Reaction

Total RNA was isolated using an RNeasy® Mini Kit (Qiagen, Düsseldorf, Germany) and a QIA-Cube automated sample preparer (Qiagen). RNA concentration was determined by UV_260 nm_ using a NanoDrop ND1000 (Thermo Scientific). cDNA synthesis was performed using a High-Capacity cDNA Reverse Transcription Kit (Life Technologies Ltd). Real-time PCR was performed using TaqMan Gene Expression Assays (Life Technologies Ltd) and TaqMan Fast Universal PCR Master Mix No AmpErase UNG (Life Technologies Ltd) on a 7900 HT Fast Real-Time PCR System with SDS 2.2 Software (Life Technologies Ltd). Measurements were performed in triplicates, using the gene for human β-actin as an endogenous control.

### Phosphatase Activity Assay

Phosphatase activity was assessed using the EnzChek® Phosphatase Assay Kit (Life Technologies Ltd) according to the manufacturer’s instructions and as described previously [18].

### Western Blot

Proteins were separated by SDS-polyacrylamide gel electrophoresis and transferred onto nitrocellulose membranes (Life Technologies Ltd). Membranes were blocked overnight with blocking solution (Tris-buffered saline containing 1% Tween 20 supplemented with 5% bovine serum albumin), and incubated with the diluted primary antibody (concentrations according to the manufactureŕs instructions) in blocking solution for an appropriate time. Membranes were washed with washing solution (blocking solution without bovine serum albumin) for 1 h, and then incubated with horseradish peroxidase (HRP)-labelled secondary anti-mouse-, anti-goat- or anti-rabbit-IgG-antibody (Santa Cruz Biotechnology, Inc., Santa Cruz, CA) diluted (1∶3000) in blocking solution for up to 30 min. Finally, membranes were washed for 1 h with washing solution and immunoreactive proteins were detected using an enhanced chemiluminescence detection kit (GE Healthcare, Little Chalfont, UK). Densitometric analysis of Western blots was performed using the National Institutes of Health (NIH) Image software.

### Small Interfering RNA (siRNA) Transfection

THP-1 cells were seeded 3 days before transfection to a density of 4×10^5^ cells per T75 flask. Three different annealed silencer pre-designed siRNA oligonucleotides targeting the *ptpn2* gene were obtained from Life Technologies Ltd. Per transfection, 100 pmol of each of the three gene specific siRNA oligonucleotides were transfected into THP-1 cells using the Amaxa nucleofector system (Lonza, Walkersville, MD) according to the manufacturer’s instructions resulting in a final siRNA concentration of 1 nMol/ml. The achieved PTPN2 knock.-down was about 70%.

After transfection, THP-1 cells were cultured in a 24-well plate for 36 h before treatment. Non-specific control siRNA (100 pmol/transfection, Life Technologies Ltd) was used as negative control.

### Enzyme-linked Immunosorbent Assay (ELISA)

Supernatants from THP-1 cell cultures were collected and stored at −80°C until further analysis. ELISA kits for detection of human IL-6, and human monocyte chemoattractant protein (MCP)-1 were obtained from R&D Systems (Minneapolis, MN). Assays were performed according to the manufacturer’s instructions. Absorbance at 450 nm was determined using a BioTek Synergy 2 Microplate reader with Gen 5 Software version 5.1.11 (BioTek Instruments, Inc., Winooski, VT). Measurements were performed in duplicates.

### Statistical Analysis

Data are presented as means ± standard deviations for a series of *n* experiments. Statistical analysis was performed by analysis of variance (ANOVA) followed by the Student–Newman–Keuls (for cell-line based experiments) or Mann-Whitney U (for mouse experiments) post hoc test. P values <0.05 were considered significant.

## Results

### Spermidine Treatment Induces PTPN2 Protein Expression in Human Monocytic THP-1 Cells

**Figure 1:**
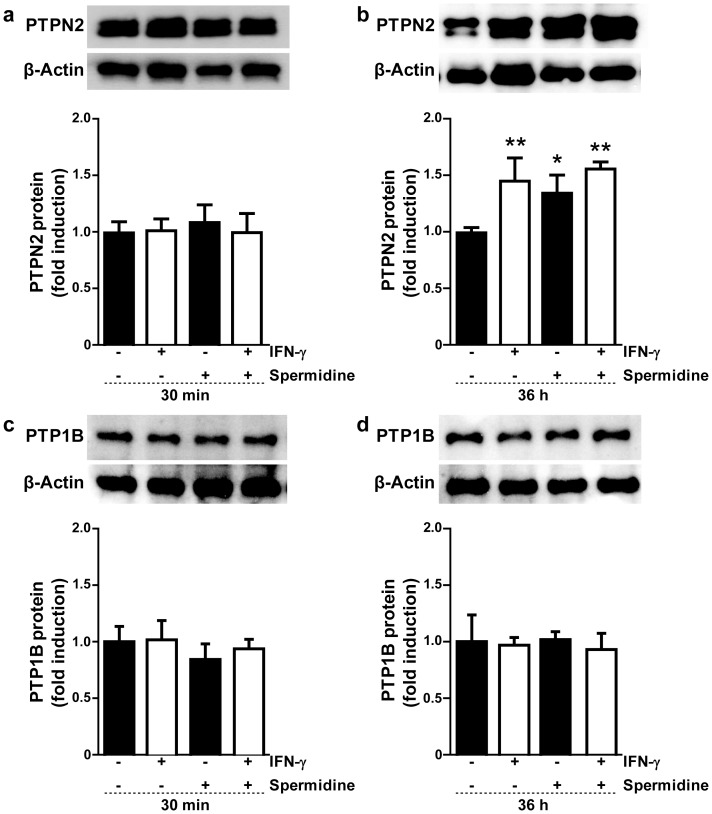
Effect of spermidine and interferon-gamma (IFN-γ) treatment on protein tyrosine phosphatases (PTPN2 and PTP1B) expression in human monocytic THP-1 cells. THP-1 cells were treated with IFN-γ (1000 U/ml) and/or spermidine (100 µM) for (**a**, **c**) 30 min or (**b**, **d**) 36 h. Whole-cell lysates were obtained and PTPN2 and PTP1B protein levels were analyzed by Western blot using β-actin as a loading control. The relative protein levels were assessed by densitometry (n = 3). Data are expressed as fold induction with respect to untreated cells. Asterisks indicate significant differences between treated and untreated cells (* = p<0.05, ** = p<0.01).

Because PTPN2 is crucially involved in negatively regulating IFN-γ induced signaling [17], we hypothesize that continuous pharmacological activation of PTPN2 by spermidine could attenuate the pro-inflammatory signaling induced by this cytokine. We thus investigated if PTPN2 activity and/or expression can be enhanced by spermidine in THP-1 monocytes and if this would attenuate the pro-inflammatory response to IFN-γ in these cells. To address the effect of spermidine on PTPN2 expression, THP-1 cells were incubated in the presence of IFN-γ and/or spermidine for 30 min or 36 h, and lysates were analyzed by Western blot using a monoclonal antibody specific for PTPN2. As shown in Figure 1a, neither IFN-γ nor spermidine treatment resulted in an increase in PTPN2 protein levels after 30 min. However, there was a significant increase in PTPN2 levels in both IFN-γ or spermidine treated cells after 36 h treatment, but co-stimulation had no additive effect (Figure 1b).

To investigate the specificity of spermidine for PTPN2, we additionally analyzed the protein expression of PTP1B, a closely related phosphatase that also regulates IFN-γ-induced responses [29]. In contrast to the observed increase in PTPN2 expression, neither IFN-γ nor spermidine treatment changed the expression levels of PTP1B (Figures 1c and 1d). Taken together, this indicates that both IFN-γ and spermidine specifically induce PTPN2 expression after 36 h treatment.

### Spermidine Treatment Increases Phosphatase Activity of PTPN2 in Human Monocytic THP-1 Cells

**Figure 2:**
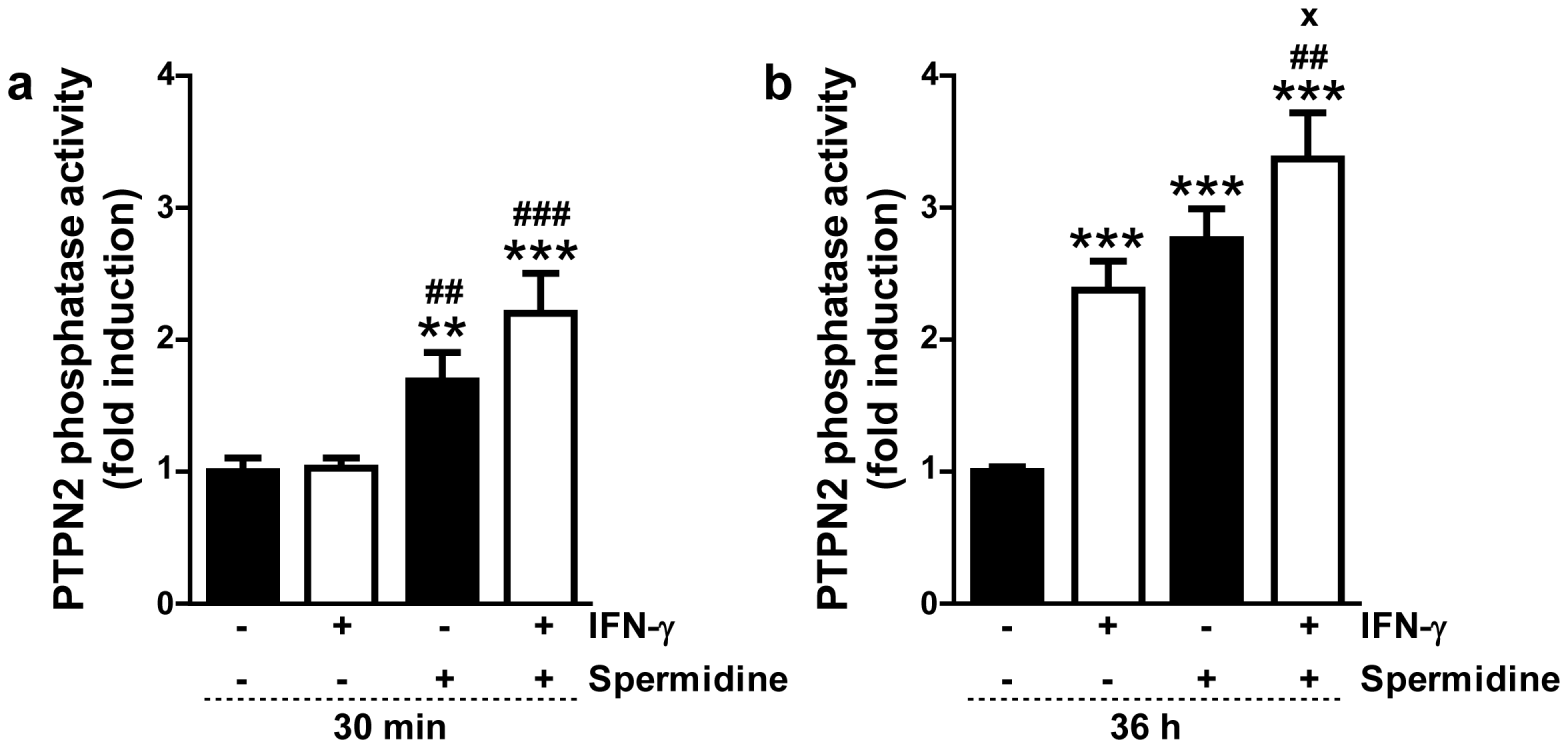
Protein tyrosine phosphatase non-receptor type 2 (PTPN2) phosphatase activity in interferon-gamma (IFN-γ)- and/or spermidine-treated human monocytic THP-1 cells. THP-1 cells were treated with IFN-γ (1000 U/ml) and/or spermidine (100 µM) for (**a**) 30 min or (**b**) 36 h. PTPN2 was immunoprecipitated from whole-cell lysates and phosphatase activity was measured (n = 3). Blots were probed for PTPN2 to show equivalent protein levels. Data are expressed as fold induction with respect to untreated cells. Significant differences compared to untreated cells (** = p<0.01, *** = p<0.001), compared to cells treated with IFN-γ only (^##^ = p<0.01, ^###^ = p<0.001), or compared to cells treated with spermidine alone (^x^ = p<0.05) are indicated.

To further explore the effects of spermidine on PTPN2 in human THP-1 cells, we measured the phosphatase activity of PTPN2 in response to treatment with IFN-γ and/or spermidine by performing immunoprecipitation of PTPN2 from whole cell lysates and analyzing its phosphatase activity using a fluorescence-based assay. As shown in Figure 2a, a significant increase in PTPN2 phosphatase activity was observed after treatment of cells for 30 min with spermidine or with IFN-γ and spermidine (p<0.01 and p<0.001, respectively), compared to untreated cells or IFN-γ-treated cells. This indicates that spermidine increases enzymatic activity of PTPN2 at a time point in which protein expression is not affected (Figure 1a). However, at this time point co-treatment with spermidine and IFN-γ did not have a statistically significant additive effect on PTPN2 activity, compared to treatment with spermidine or IFN-γ alone. When cells were incubated for 36 h, IFN-γ alone was able to significantly increase the phosphatase activity of PTPN2 (p<0.001; Figure 2b), which is consistent with our previous results [18]. Moreover, cells treated with spermidine alone showed significantly increased PTPN2 activity at 36 h, with nearly a 3-fold induction compared to untreated cells (p<0.001, Figure 2b). The co-treatment of THP-1 cells with both IFN-γ and spermidine for 36 h significantly further increased PTPN2 phosphatase activity, compared to either IFN-γ or spermidine alone (p<0.01 and p<0.05, respectively; Figure 2b). This observation clearly indicates an additive effect of both stimuli at this time point with respect to PTPN2 phosphatase activity, effect that was not observed on protein expression (Figure 1b). Because phosphatase activity was normalized to the PTPN2 protein content of the cells, the observed increase in activity is not due to the increase in PTPN2 protein level, but rather to actual activation of the available PTPN2 protein, and appears to occur independently of the increase in PTPN2 expression levels.

### Repetitive Administration of Spermidine Causes Persistent PTPN2 Activation in Human Monocytic THP-1 Cells

**Figure 3:**
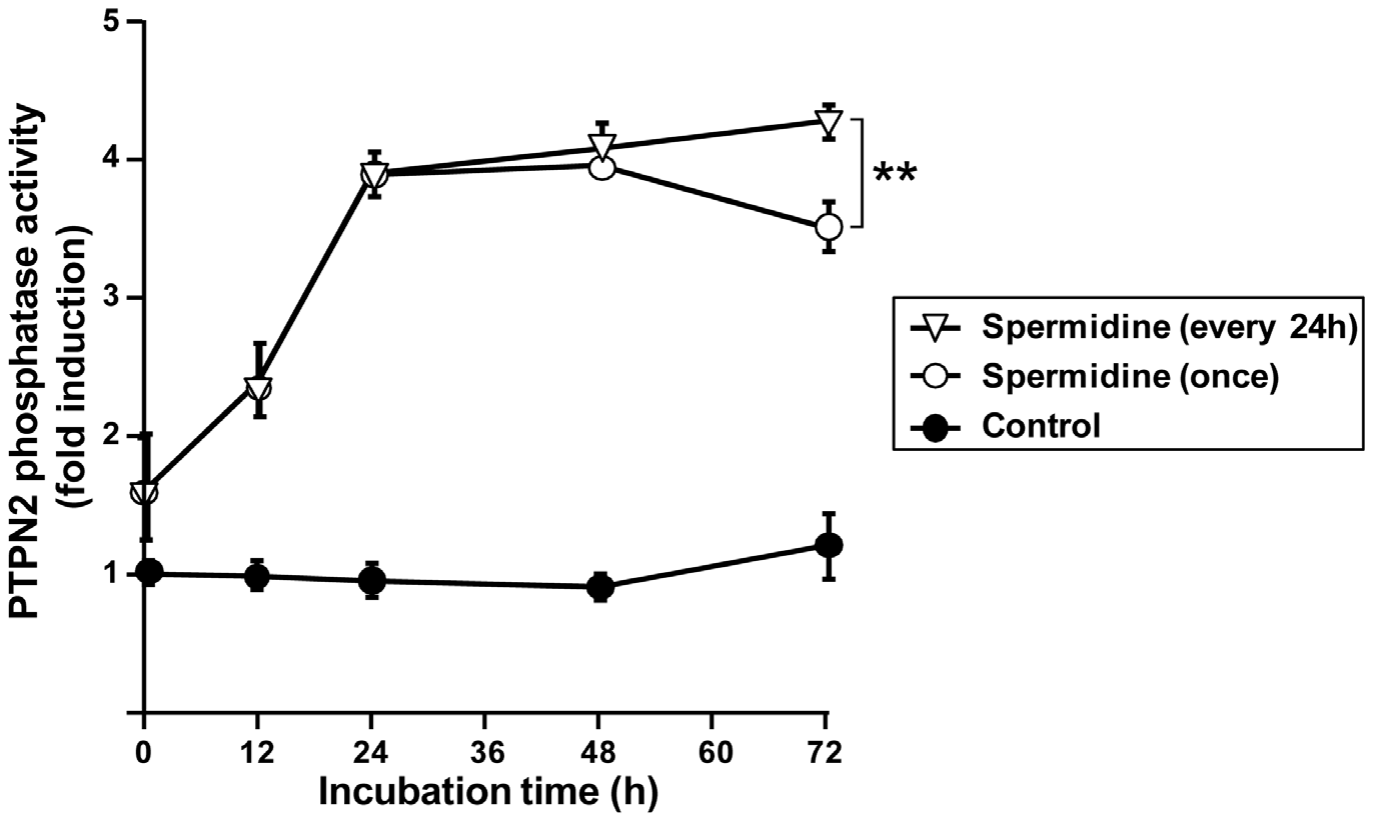
Changes in protein tyrosine phosphatase non-receptor type 2 (PTPN2) phosphatase activity after spermidine treatment of human monocytic THP-1 cells. THP-1 cells were incubated with (white symbols) or without (black symbols) spermidine (100 µM) for 72 hours. For spermidine treated cells, the polyamine was either added once at 0 h (white circles) or every 24 h (white triangles). PTPN2 was immunoprecipitated from whole-cell lysates, and phosphatase activity was measured (n = 3). Data are expressed as fold induction with respect to untreated cells. Asterisks indicate a significant difference between phosphatase activity at the 72 h time point for the two spermidine treatments (** = p<0.01).

**Figure 4:**
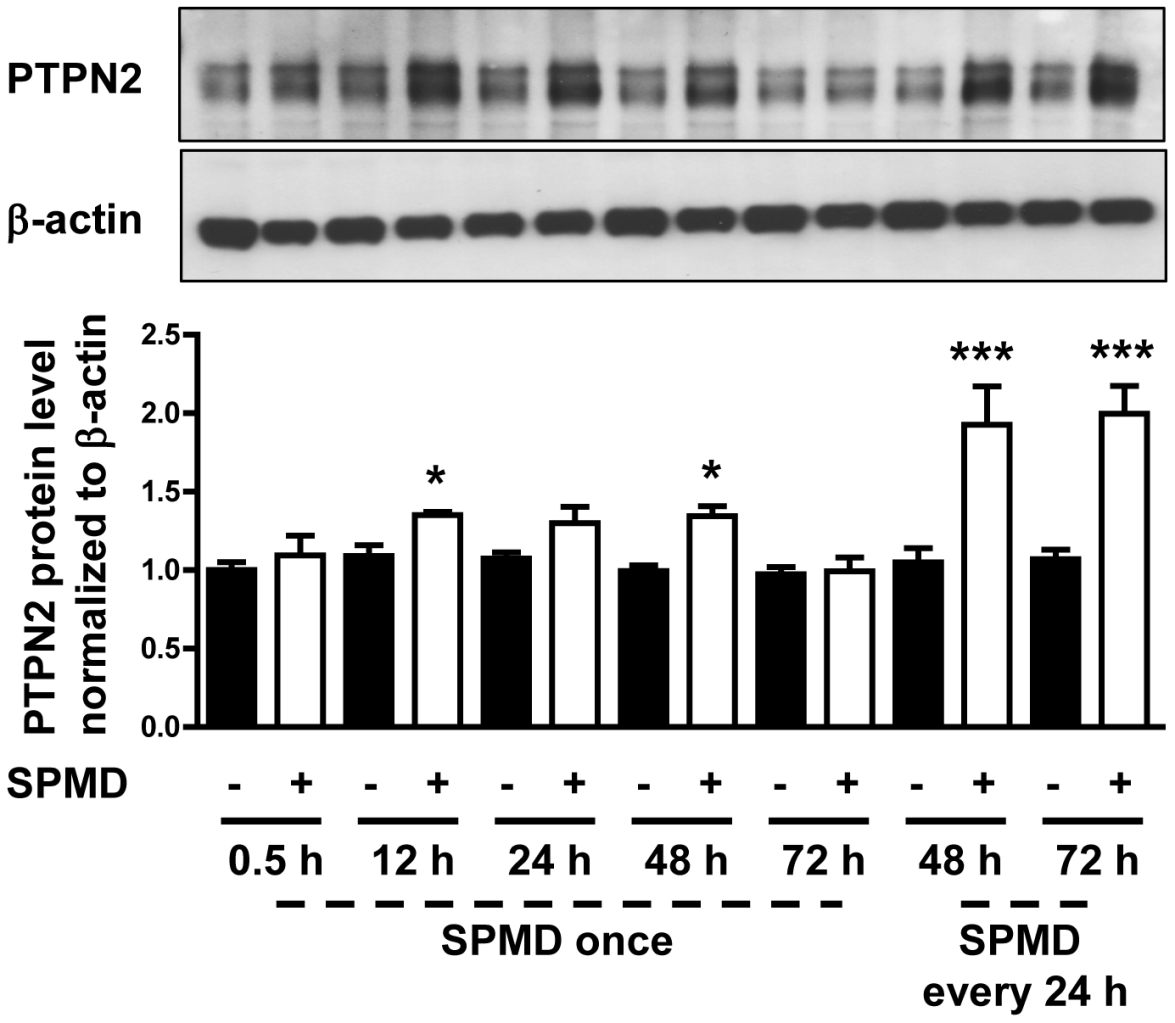
THP-1 cells were incubated with (white bars) or without (black bars) spermidine (100 µM) for 72 hours. For spermidine treated cells, the polyamine was either added once at 0 h or every 24 h as indicated. The cells were harvested at the indicated time points and analyzed for PTPN2 expression (n = 3). Significant differences from the respective controls are denoted by asterisks (* = p<0.05, *** = p<0.05).

Because spermidine may be metabolized by the cells and disappear rapidly from the media, and in order to explore the effects of dosing intervals for future preclinical investigations, we measured the change in phosphatase activity of PTPN2 over time after a single dose or after multiple doses of spermidine in THP-1 cells. Cells were incubated with spermidine for up to 72 h, adding the polyamine either once at 0 h or three times at intervals of 24 h. Phosphatase activity was measured at 0 h, 12 h, 24 h, 48 h, and 72 h. As shown in Figure 3, PTPN2 phosphatase activity reached the maximum value (∼4-fold the basal value) 24 h after spermidine treatment. Even without further spermidine treatment, PTPN2 activity was maintained at this maximum level for an additional 24 h. However, whereas the effects of a single dose of spermidine were observed to wear off between 48 and 72 h, dosing of additional spermidine every 24 h was effective, with even a slight further increase in PTPN2 activity after each spermidine administration over the 72 h testing period. We then measured PTPN2 protein at 48 and 72 h in response to spermidine treatment of THP-1 cells. We found that the spermidine-induced increase in PTPN2 enzymatic activity is reflected by an increase in spermidine-induced PTPN2 protein level over time. Interestingly, repeated treatment with spermidine (every 24 h) causes a further increase in PTPN2 protein level compared to single spermidine treatment (Figure 4). These findings indicate that administration of repetitive doses of spermidine every 24 to 48 h was effective in maintaining PTPN2 activity and even further induced PTPN2 protein level. This dosing schedule is a reasonable starting point for performing pharmacokinetic and pharmacodynamic preclinical studies to determine the appropriate clinical dosing schedule.

### PTPN2 Activation by Spermidine Ameliorates IFN-γ-induced Phosphorylation of STAT1, STAT3 and p38

**Figure 5:**
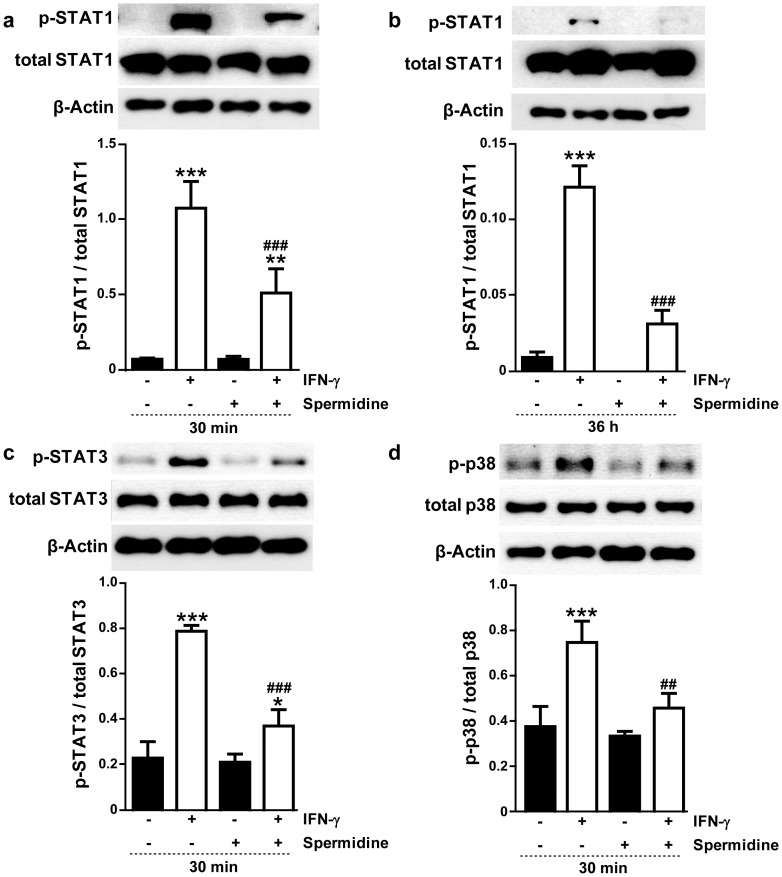
Effects of spermidine treatment on the phosphorylation levels of signal transducers and activators of transcription (STATs) and p38 mitogen-activated protein kinase (MAPK) in interferon-gamma (IFN-γ)-treated THP-1 cells. Representative Western blots and densitometry (n = 3) show phosphorylation status of (**a** and **b**) STAT1, (**c**) STAT3 and (**d**) p38 MAPK in THP-1 cells treated with IFN-γ (1000 U/ml) and/or spermidine (100 µM) for (**a**, **c**, and **d**) 30 min or (**b**) 36 h. Blots were probed for β-actin to show equal protein loading. Significant differences compared to untreated cells (* = p<0.05, ** = p<0.01, *** = p<0.001) or compared to cells treated with IFN-γ only (^##^ = p<0.01, ^###^ = p<0.001) are indicated.

After demonstrating that spermidine is able to induce PTPN2 protein expression and phosphatase activity in THP-1 monocytes, we next studied the functional relevance of this observation. Previous studies found STAT1, STAT3, and p38 to be PTPN2 dephosphorylation targets [14], [15], [16], [30], and indicated that loss of PTPN2 activity causes increased activation of STAT1, STAT3, and p38 in IFN-γ-treated THP-1 cells [17]. Thus, we explored whether the induction of the expression and activity of PTPN2 by spermidine results in reduced activation (as monitored by phosphorylation status) of its targets STAT1, STAT3 and p38. As expected, treatment of THP-1 cells with IFN-γ for 30 min markedly increased the phosphorylation of STAT1 (p<0.001; Figure 5a). Co-administration of spermidine significantly reduced IFN-γ-induced STAT1 phosphorylation (p<0.001). Similar results were found for STAT1 in cells treated for 36 h (Figure 5b), with significantly increased phosphorylation of STAT1 in cells treated with IFN-γ (p<0.001), which was reduced upon co-treatment with spermidine (p<0.001). Comparable findings were also observed for STAT3 and p38 phosphorylation for cells treated with spermidine for 30 min. As shown in Figure 5c and 5d, IFN-γ increased phosphorylation of both STAT3 and p38 (p<0.001). Spermidine administration diminished the phosphorylation of STAT3 and p38 induced by IFN-γ in THP-1 cells compared to cells only treated with IFN-γ (p<0.001 and p<0.01, respectively). These findings demonstrate that PTPN2 activation by spermidine is able to reduce downstream pro-inflammatory signal transduction events induced by IFN-γ in human THP-1 monocytes.

### PTPN2 Activation by Spermidine Reduces Secretion and Expression of STAT1 and p38-MAPK-dependent Pro-inflammatory Cytokines in THP-1 Cells

**Figure 6:**
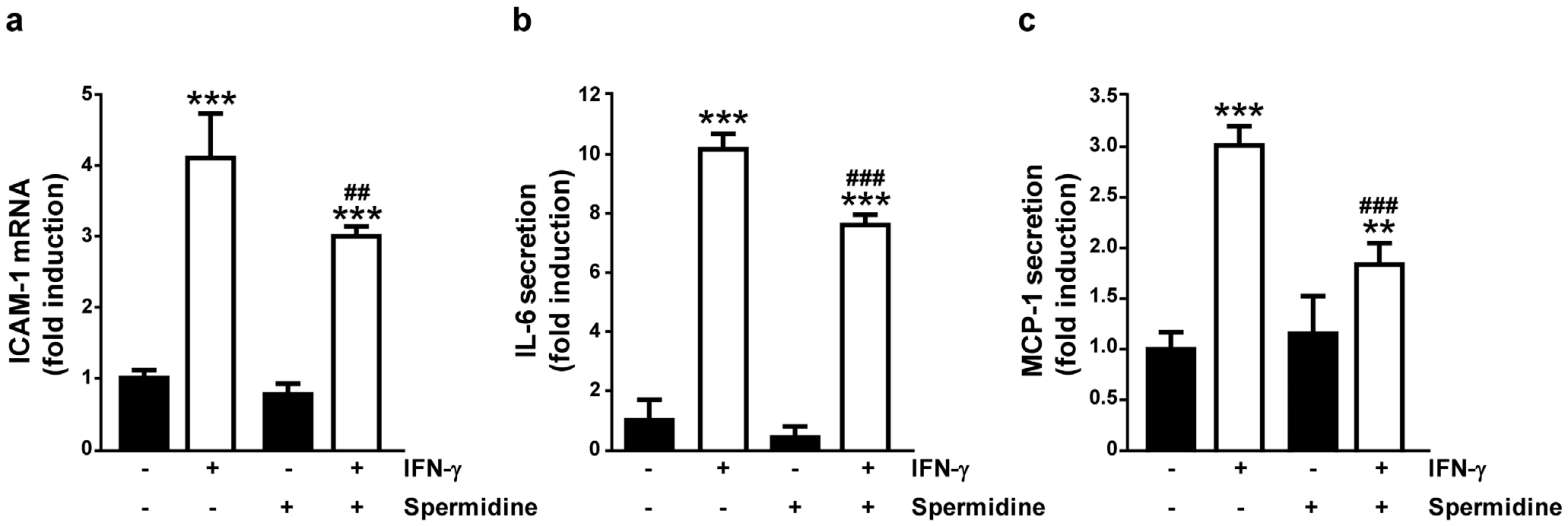
Cytokine signaling in interferon-gamma (IFN-γ)- and/or spermidine-treated THP-1 cells. The graphs show (**a**) mRNA levels of intracellular cell adhesion molecule (ICAM)-1, (**b**) secretion of interleukin (IL)-6, and (**c**) secretion of monocyte chemoattractant protein (MCP)-1 in THP-1 cells treated with IFN-γ (1000 U/ml) and/or spermidine (100 µM) for 24 h (n = 3). Data are expressed as fold induction with respect to untreated cells. Significant differences compared to untreated cells (** = p<0.01, *** = p<0.001) or compared to IFN-γ-treated cells (^##^ = p<0.01, ^###^ = p<0.001) are indicated.

To determine the effects of spermidine on the activation of IFN-γ-induced signal transducers as well as on the synthesis of IFN-γ-induced cytokines, we next measured the gene expression and secretion of STAT1 and p38-MAPK-dependent cytokines in THP-1 cells. As shown in Figure 5a and consistent with previous results [17], IFN-γ treatment significantly increased mRNA expression of intracellular cell adhesion molecule (ICAM)-1 (p<0.001). Spermidine co-treatment for 24 h reduced IFN-γ-stimulated mRNA expression of ICAM-1 (p<0.01; Figure 6a), whereas spermidine treatment alone had no significant effect on ICAM-1 mRNA levels (Figure 6a). We then tested whether PTPN2 activation by spermidine alters the amount of cytokines secreted by THP-1 cells. As shown in Figure 6b, incubation for 24 h with IFN-γ markedly induced the secretion of IL-6 (p<0.001). This effect was clearly reduced after spermidine co-treatment of cells (p<0.001). Similarly, spermidine was able to ameliorate the IFN-γ-induced secretion of MCP-1 from THP-1 cells (p<0.001; Figure 6c). These data demonstrate that spermidine treatment reduces pro-inflammatory gene expression and cytokine secretion induced by IFN-γ in THP-1 cells and support the hypothesis of an anti-inflammatory effect of spermidine.

### Anti-inflammatory Effects of Spermidine are Dependent on PTPN2

**Figure 7:**
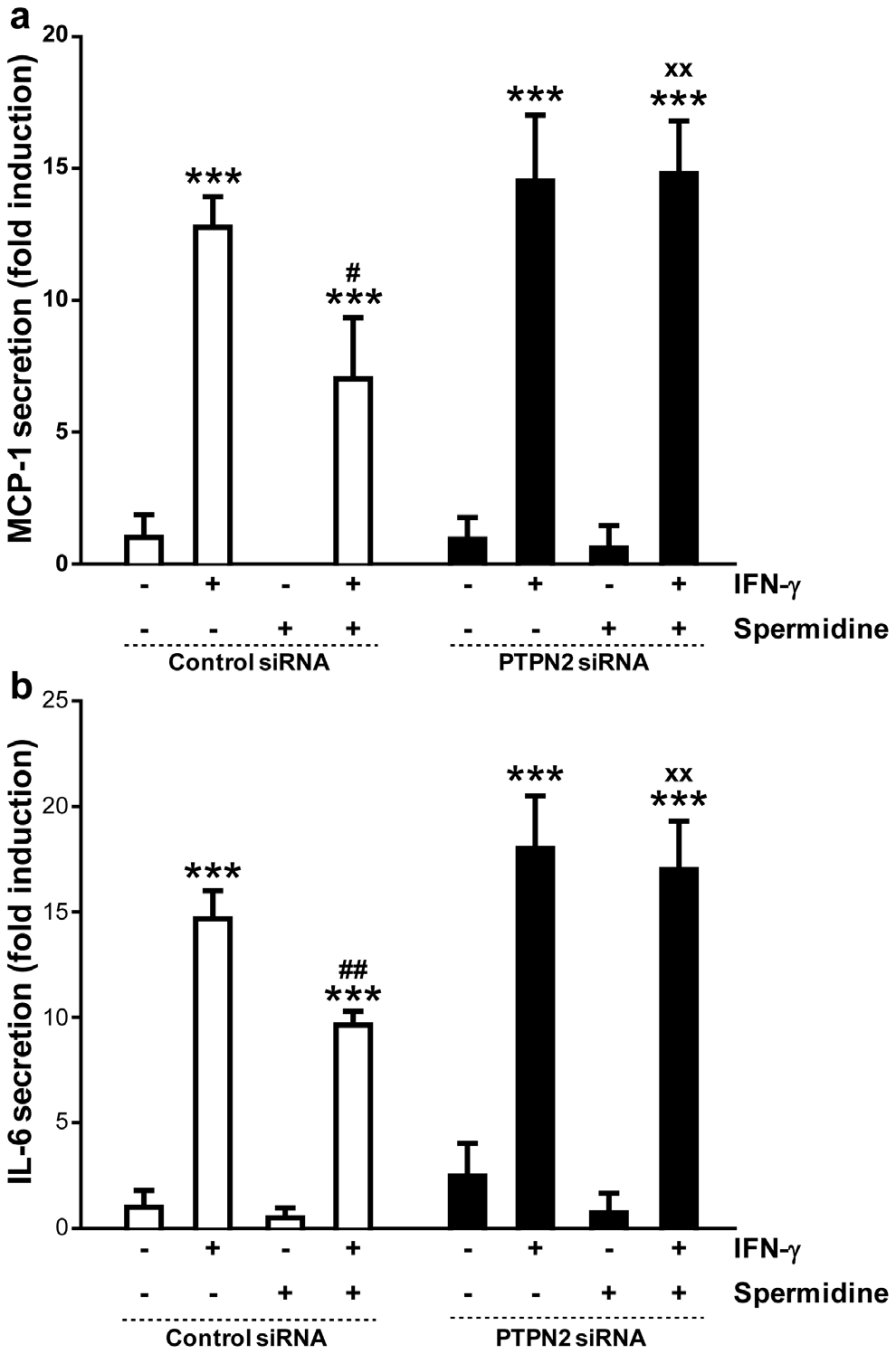
Effect of protein tyrosine phosphatase non-receptor type 2 (PTPN2) knockdown on cytokine signaling in human monocytic THP-1 cells treated with interferon-gamma (IFN-γ) and/or spermidine. THP-1 cells were transfected with either nonspecific small interfering RNA (siRNA) or *ptpn2* siRNA. The graphs show secretion levels of (**a**) monocyte chemoattractant protein (MCP)-1, and (**b**) interleukin (IL)-6 in THP-1 cells treated with IFN-γ (1000 U/ml) and/or spermidine (100 µM) for 24 h (n = 3). Data are expressed as fold induction respect to untreated cells transfected with non-specific siRNA. Significant differences compared to untreated (*** = p<0.001), IFN-γ-treated (^#^ = p<0.05, ^##^ = p<0.01), or IFN-γ and spermidine co-treated (^xx^ = p<0.01) cells transfected with non-specific siRNA are indicated.

To confirm that the decrease in the secretion of IFN-γ-induced cytokines after spermidine treatment was mediated by PTPN2, we quantified the levels of MCP-1 and IL-6 secreted by PTPN2-knockdown cells treated with IFN-γ and/or spermidine. THP-1 monocytes were transfected with either siRNA oligonucleotides targeting *ptpn2* or with nonspecific siRNA sequences as a control, and treated with IFN-γ and/or spermidine for 24 h. As shown in Figure 7a, spermidine treatment markedly reduced the IFN-γ-induced secretion of MCP-1 in cells transfected with non-specific siRNA (p<0.05), whereas no significant reduction was observed in PTPN2-knockdown cells treated with spermidine and IFN-γ. Additionally, incubation of cells with spermidine significantly reduced IL-6 secretion induced by IFN-γ in cells transfected with non-specific siRNA (p<0.01), whereas this effect was absent in PTPN2-deficient cells (Figure 7b). Taken together, these results show that the observed reduction of IFN-γ-induced cellular responses by spermidine treatment of THP-1 cells is mediated by PTPN2.

### Spermidine Treatment Reduces Weight Loss and Ameliorates Mucosal Damage in Mice with DSS-induced Colitis

To investigate the anti-inflammatory potential of spermidine *in vivo*, acute colitis was induced in mice by adding 2.5% DSS in the drinking water. During the induction of colitis, one group of mice was treated daily with spermidine by oral gavage, while the other group received oral gavages with water. Control groups received no DSS and oral gavages with either spermidine or water.

**Figure 8:**
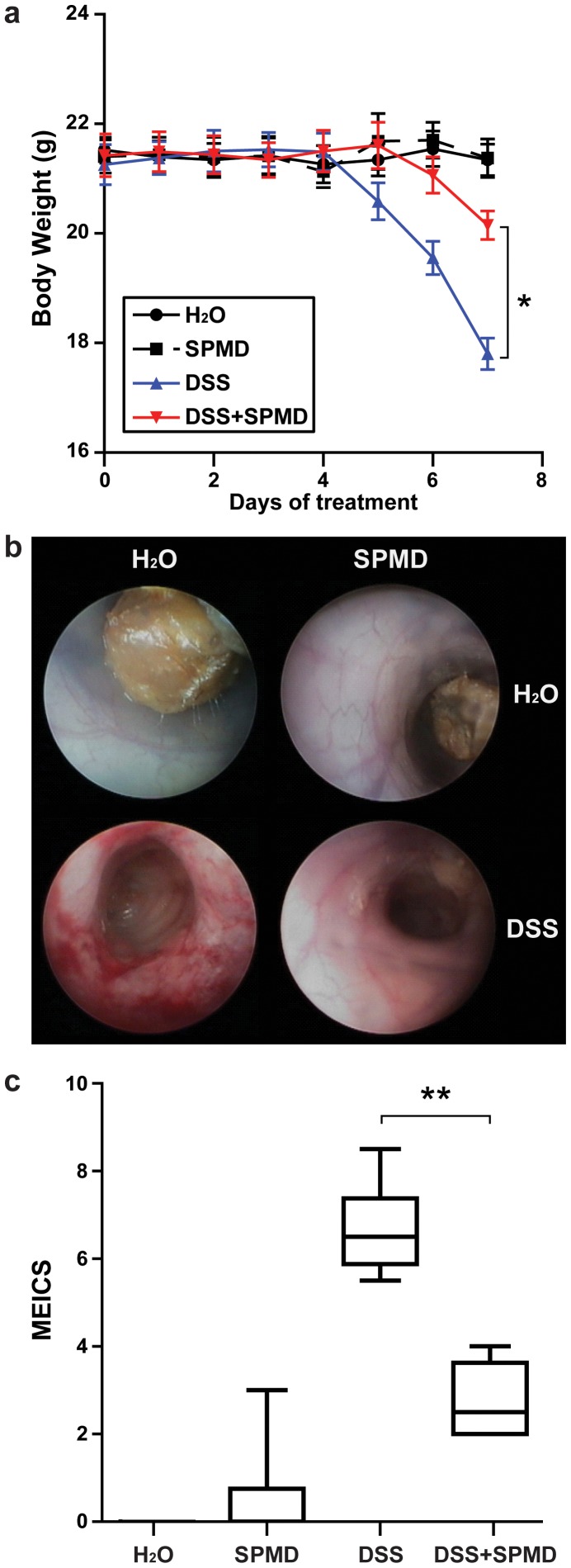
Effects of spermidine treatment on weight curves and endoscopic colonic damage in mice with DSS-induced acute colitis. Female C57BL/6J-Clr mice received water (H_2_O), 150 µl 0.1 M spermidine solution (SPMD), 2.5% DSS or 2.5% DSS plus 150 µl of 0.1 M spermidine solution for 8 days. The panels show (**a**) weight curves during colitis induction, (**b**) representative pictures of colonoscopy performed on day 8, and (**c**) colonoscopic scoring. Results are representative of two independent experiments with 6 mice in each group. Asterisks indicate significant differences as assessed by Mann-Whitney U post hoc test (* = p<0.05, ** = p<0.01).

As expected, DSS treatment induced a significant weight loss when compared to that in water-fed mice (Figure 8a). While spermidine treatment had no effect on body weight in water-fed mice, it significantly reduced weight loss in DSS-treated mice (p<0.05; Figure 7a). To assess whether the differences in weight loss correlated with macroscopic signs of inflammation, colonoscopy was performed on day 8 of DSS treatment (Figure 8b), and the results were evaluated using MEICS score (Figure 8c). As shown in Figure 8b, no signs of inflammation could be detected in control animals and solid stool pellets were visible. In DSS-treated mice however, thickened mucosa with granular appearance and non-transparent bowel wall, altered or even invisible vascular pattern with occasional bleeding, and smeary stool was observed, indicative for severe mucosal inflammation. Spermidine treatment alone did not change the macroscopic appearance of the bowel in water-fed mice. In DSS-treated mice however, spermidine administration reduced the severity of inflammation: when compared to mice receiving only DSS, the loss of transparency of the bowel wall was less pronounced with reduced granularity, the vascular pattern appeared more normal and no bleeding was detectable (Figure 8b). Evaluation of colonoscopic results using MEICS score showed a significant reduction of DSS-induced intestinal damage in mice treated with spermidine compared to those treated with water (p<0.01; Figure 8c). This indicates that spermidine treatment reduces DSS-induced weight loss and colonic damage in mice.

### Spermidine Administration Decreased DSS Mediated Inflammatory Infiltration and Epithelial Damage in Mouse Colon

**Figure 9:**
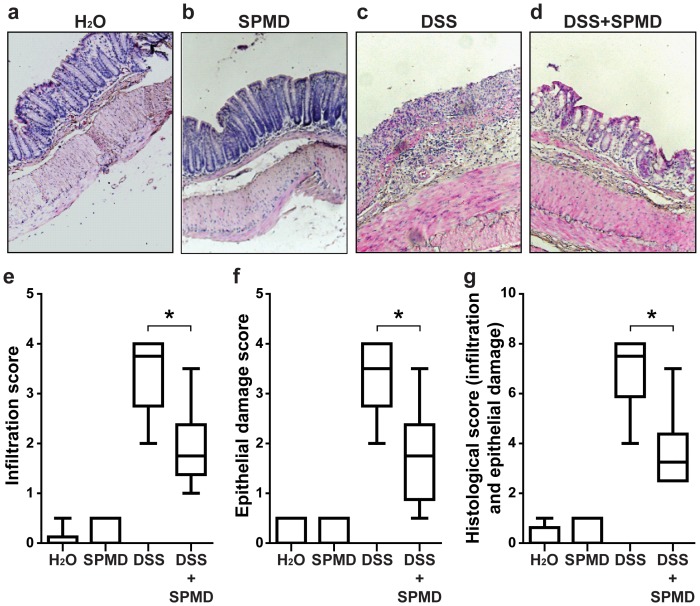
Effects of spermidine on epithelial damage and inflammation in mice with DSS-induced acute colitis. Female C57BL/6J-Clr mice received water (**a**; H_2_O), 150 µl of 0.1 M spermidine solution (**b**; SPMD), 2.5% DSS (**c**) or 2.5% DSS plus 150 µl of 0.1 M spermidine solution (**d**) for 8 days. Panels a, b, c, and d show representative pictures from H&E stained histological sections. The graphs show severity of inflammatory cell infiltration (**e**), epithelial damage (**f**) or combined scoring (**g**). Results are representative of two independent experiments with 6 mice in each group. Asterisks indicate significant differences as assessed by Mann-Whitney U post hoc test (* = p<0.05).

**Figure 10:**
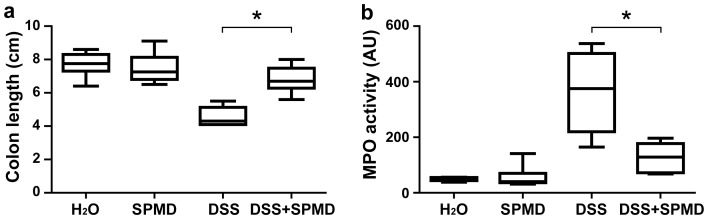
Indicators of inflammation in mice with DSS-induced acute colitis, which were treated or not with spermidine. Female C57BL/6J-Clr mice received water (H_2_O), 150 µl of 0.1 M spermidine solution (SPMD), 2.5% DSS or 2.5% DSS plus 150 µl of 0.1 M spermidine solution for 8 days. The graphs depict (**a**) colon length and (**b**) MPO activity at day 8. Results are representative of two independent experiments with 6 mice in each group. Asterisks indicate significant differences as assessed by Mann-Whitney U post hoc test (* = p<0.05).

To assess the extent of microscopic damage, histological analysis of colonic tissue specimen was performed. The mucosa of mice receiving either water or spermidine, showed regular crypt architecture and no signs of inflammation (Figures 9a and b). As expected, DSS treatment induced a severe inflammatory response with an almost complete loss of crypts, strong infiltration of lymphocytes in both mucosa and submucosa, and thickening of the bowel wall (Figure 9c). In DSS-treated mice receiving spermidine, however, the loss of crypts was less pronounced and infiltration of inflammatory cells was reduced when compared to mice receiving DSS alone (Figures 9c and 9d). Histological scoring for inflammatory infiltrates and epithelial damage corroborated these findings (Figures 9e–g), indicating that spermidine treatment reduces epithelial damage and inflammatory infiltration in mice with DSS induced colitis. Further, we assessed additional indicators of colitis such as colon length and MPO activity. DSS treatment induced a shortening of the colon in both spermidine treated and non-treated animals, however the shortening was significantly less pronounced in mice receiving spermidine (Figure 10a). In addition, the DSS-induced increase in MPO activity, a marker of neutrophil infiltration, was significantly lower in mice treated with spermidine compared to those treated with water (p<0.05; Figure 10b). Taken together, these results indicate that spermidine treatment ameliorates DSS-induced intestinal damage in mice.

## Discussion

In the last decade, our understanding of the pathophysiological mechanisms of IBD, as well as the immunologic and genetic factors involved, has improved considerably. These advances have resulted in the development of new treatments for patients suffering from IBD [31]. However, since this new therapeutics, mainly biological, often feature severe side effects, the need for well-tolerable, but nevertheless effective treatment options is obvious. Here, we have demonstrated that pharmacological activation of PTPN2 by the polyamine spermidine ameliorates inflammatory responses both in an *in vitro* as well as in an *in vivo* model of inflammation. Our data have shown that spermidine activates PTPN2, which plays a crucial role in controlling pro-inflammatory signaling and gene expression events. Subsequently, activation of PTPN2 results in decreased activation of pro-inflammatory STAT1, STAT3 and p38 signal transmitters as well as in decreased secretion of the pro-inflammatory cytokines IL-6 and MCP-1 in human monocytes. Even more interestingly, administration of spermidine in a mouse model of colitis results in a significant reduction in the disease severity, thereby validating spermidine as a plausible new therapy option for the treatment of IBD. Moreover, because spermidine is present in a broad variety of foods and is also synthesized endogenously, its administration seems to be a well-tolerable treatment option.

IFN-γ is the prototypical macrophage-activating factor. In the classical signaling pathway, IFN-γ initiates its signal transduction cascade via tyrosine phosphorylation of STAT1. Subsequently, phosphorylated STAT1 dimerizes and the dimers bind to IFN-γ-stimulated response elements, inducing the transcription of various genes [32]. We have demonstrated that spermidine treatment markedly reduces IFN-γ-induced phosphorylation of STAT1, which leads to reduced secretion of the cytokine MCP-1 in a PTPN2-dependent manner.

Besides the classical STAT1 signaling cascade, IFN-γ activates several other downstream pathways, including the p38 MAPK signaling cascade [32]. One of the immune targets regulated by the p38 MAPK signaling pathway is IL-6, a potent cytokine that regulates immune and inflammatory responses. Due to its key role in the pathogenesis of IBD, therapies targeting this cytokine are in development [33]. We have found that spermidine treatment significantly decreased the IFN-γ-induced phosphorylation of p38 MAPK in a PTPN2-dependent manner. These results are in accordance with previous data showing that PTPN2 negatively regulates the IFN-γ-induced signaling via p38 MAPK, including IL-6 secretion [17]. Correlating with the decreased levels of IL-6, we found reduced phosphorylation of STAT3 as well as lowered mRNA expression of ICAM-1, both downstream molecules of the p38 MAPK signaling cascade [34], [35], in THP-1 cells treated with spermidine and IFN-γ compared to cells that were only treated with IFN-γ.

Both IL-6 and MCP-1 levels are increased in the intestinal mucosa of IBD patients [36]. While IL-6 signaling is involved in shaping the adaptive immune response [37], MCP-1 promotes monocyte infiltration into inflamed tissues. In the gut MCP-1 also inhibits the differentiation of monocytes into mature intestinal macrophages (IMACs) with attenuated immune functions. In active IBD, an increased fraction of over-activated and hyper-reactive IMACs has been reported [38], [39], [40], which results in an increased production of pro-inflammatory cytokines. Additionally, elevated levels of MCP-1 can be found in the intestinal mucosa of IBD patients. Therefore, reduced MCP-1 production upon spermidine treatment might reduce the attraction of inflammatory cells into the intestine and thereby decrease inflammatory responses. Consistent with this hypothesis, spermidine treatment also significantly reduced the number of inflammatory cells infiltrating the mucosa of DSS treated mice.

In addition to the reduction of inflammatory cell infiltration into the mucosa, spermidine treatment also significantly reduced epithelial damage during DSS-mediated colitis. This might partially be due to the fact that PTPN2, in addition to its regulatory role on STAT and MAPK signaling in immune cells, also plays an important protective role in intestinal epithelial cells, maintaining barrier function and homeostasis during inflammation [18], [19], [25].

All cells contain substantial amounts of either spermidine or one of the other polyamines, putrescine or spermine, which are required for cell growth and proliferation [21], [22]. Although diet is the main source of polyamines, they can also be synthesized endogenously. Interestingly, reduced levels of polyamines were found in colonic samples from mice with induced chronic colitis [41]. Moreover, lowered levels of ornithine decarboxylase, the limiting enzyme in endogenous synthesis of polyamines, have been reported in colonic mucosa from IBD patients [42], suggesting that the lack of polyamines in inflamed tissue may aggravate the disease. Our results support this notion, since spermidine supplementation was able to significantly reduce inflammatory responses in both an *in vitro* model of immune cell activation and an *in vivo* model of intestinal inflammation.

In conclusion, we have shown that spermidine was able to reduce inflammation by increasing the expression and activity of PTPN2 in human THP-1 monocytes. As a consequence, PTPN2 negatively regulated the IFN-γ-induced signaling pathways via dephosphorylation of STAT1 and p38 MAPK, resulting in a decrease in the secretion of pro-inflammatory cytokines. In mice with DSS-induced acute colitis, spermidine treatment was able to reduce disease severity, supporting the anti-inflammatory potential of spermidine supplementation *in vivo*. Taken together, these findings suggest that pharmacological activation of PTPN2 by spermidine may represent a viable therapy to treat IBD and other diseases featuring chronic intestinal inflammation. Further studies to elucidate the clinical implications of these findings are therefore warranted.
